# Defective Interfering Particles with Broad-Acting Antiviral Activity for Dengue, Zika, Yellow Fever, Respiratory Syncytial and SARS-CoV-2 Virus Infection

**DOI:** 10.1128/spectrum.03949-22

**Published:** 2022-11-29

**Authors:** Min-Hsuan Lin, Dongsheng Li, Bing Tang, Li Li, Andreas Suhrbier, David Harrich

**Affiliations:** a Program of Infection and Inflammation, QIMR Berghofer Medical Research Institute, Herston, Queensland, Australia; b Australian Institute for Bioengineering and Nanotechnology, the University of Queensland, St. Lucia, Queensland, Australia; c Australian Infectious Disease Research Centre, GVN Center of Excellence, Brisbane, Queensland, Australia; Institute of Microbiology, Chinese Academy of Sciences

**Keywords:** dengue virus, SARS-CoV-2, coronavirus, Zika virus, yellow fever virus, respiratory syncytial virus, antiviral therapy, defective interfering particle, RNA, defective viral genome, chromatography, purification

## Abstract

More than 100 arboviruses, almost all of which have an RNA genome, cause disease in humans. RNA viruses are causing unprecedented health system challenges worldwide, many with little or no specific therapies or vaccines available. Certain species of mosquito can carry dengue virus (DENV), Zika virus (ZIKV) and yellow fever virus (YFV), where co-infection of these viruses has occurred. Here, we found that purified synthetic defective interfering particles (DIPs) derived from DENV type 2 (DENV-2) strongly suppressed replication of the aforementioned viruses, respiratory syncytial virus (RSV) and also the novel emerging virus SARS-CoV-2 in human cells. DENV DIPs produced in bioreactors, purified by column chromatography, and concentrated are virus-like particles that are about half the diameter of a typical DENV particle, but with similar ratios of the viral structural proteins envelope and capsid. Overall, DIP-treated cells inhibited DENV, ZIKV, YFV, RSV, and SARS-CoV-2 by at least 98% by mechanisms which included interferon (IFN)-dependent cellular antiviral responses.

**IMPORTANCE** DIPs are spontaneously derived virus mutants with deletions in genes that block viral replication. DIPs play important roles in modulation of viral disease, innate immune responses, virus persistence and virus evolution. Here, we investigated the antiviral activity of highly purified synthetic DIPs derived from DENV, which were produced in bioreactors. DENV DIPs purified by column chromatography strongly inhibited five different RNA viruses, including DENV, ZIKV, YFV, RSV, and SARS-CoV-2 in human cells. DENV DIPs inhibited virus replication via delivery of a small, noninfectious viral RNA that activated cellular innate immunity, resulting in robust type 1 interferon responses. The work here presents a pathway for DIP production which is adaptable to Good Manufacturing Practice, so that their preclinical testing should be suitable for evaluation in subjects.

## INTRODUCTION

Dengue virus (DENV) is a flavivirus with a small, positive-stranded RNA genome. There are four serotypes, DENV-1 to -4, all of which are transmitted by mosquitoes and cause ~100 million clinical infections annually, about half a million hospitalizations, and 25,000 deaths. Currently, antiviral drugs are not available for clinical use, but recent reports of new and repurposed drugs are encouraging (1, 2). The first licensed dengue vaccine is available, but its use and effectiveness are limited. The current standard of care for DENV-infected patients includes monitoring and replacement of fluids as required, so the repurposing of dugs (2), discovery and development of new antiviral compounds (1), and new treatment modalities (3) for DENV infection are urgent needs.

Defective interfering particles (DIPs) are a specialized type of virus-like particle (VLP) which contains a defective viral genome (DVG) (4). DIPs have the potential to be used as a novel antiviral therapy (5–8). DIPs arise naturally following viral infection of a host and they influence viral pathogenesis through mechanisms including viral interference, persistence, and immune stimulation (9). The interference of infectious virus replication involves multiple mechanisms of action which include competition of the viral genome and DVG for the viral and cellular resources required for viral genome replication, interference with viral genome packaging in newly made virions, and stimulation of interferon (IFN)-dependent cellular antiviral responses (8, 10–12). Anti-viral DIPs against influenza A virus (IAV), which have one defective interfering (DI) RNA (i.e., the DVG) called DI-244 and another called OP7, potently inhibit IAV in animal models of lethal IAV infection (13–16). Interestingly, IAV DIPs could also block replication of SARS-CoV-2 in susceptible human cell lines, where inhibition of virus was dependent on janus kinase/signal transducers and activators of transcription (JAK/STAT) signaling (12). Methods to produce DIPs in the absence of infectious virus have been reported and represent an important achievement as the potential clinical applications of DIPs are advanced (17–19).

Recently, our group reported DIPs derived from DENV which have pan-subtype anti-DENV activity *in vitro* (20). The infectious, virus-free DENV DIPs were produced by a stably transduced HEK293T cell line. The strategy to produce DENV DIPs utilized two lentiviral vectors and one retroviral vector, which were used to express the DENV structural proteins, nonstructural proteins, and a 290-nt long DI RNA (DI-290). The cell line, called HEK-DI-290-ORF, secreted DIPs in the culture supernatant, and these were partially purified by ceramic hydroxyapatite (CHT) chromatography. In this study, stir cell bioreactors were used to produce DIPs in serum-free medium (SFM) that could be purified from culture supernatant. We show that highly purified and biologically active DENV DIPs obtained by column chromatography removed >99.9% of contaminating proteins. Moreover, our results show that the purified DIPs suppress the replication of DENV, Zika virus (ZIKV), yellow fever virus (YFV), respiratory syncytial virus (RSV), and severe acute respiratory syndrome coronavirus 2 (SARS-CoV-2) omicron variant. The inhibitory effect of DENV DIPs may be due to their ability to rapidly stimulate a host innate immune response.

## RESULTS

### Compounds with PKC agonist activity increased production of DENV DIPs by HEK-DI-290-ORF cells.

**FIG 1:**
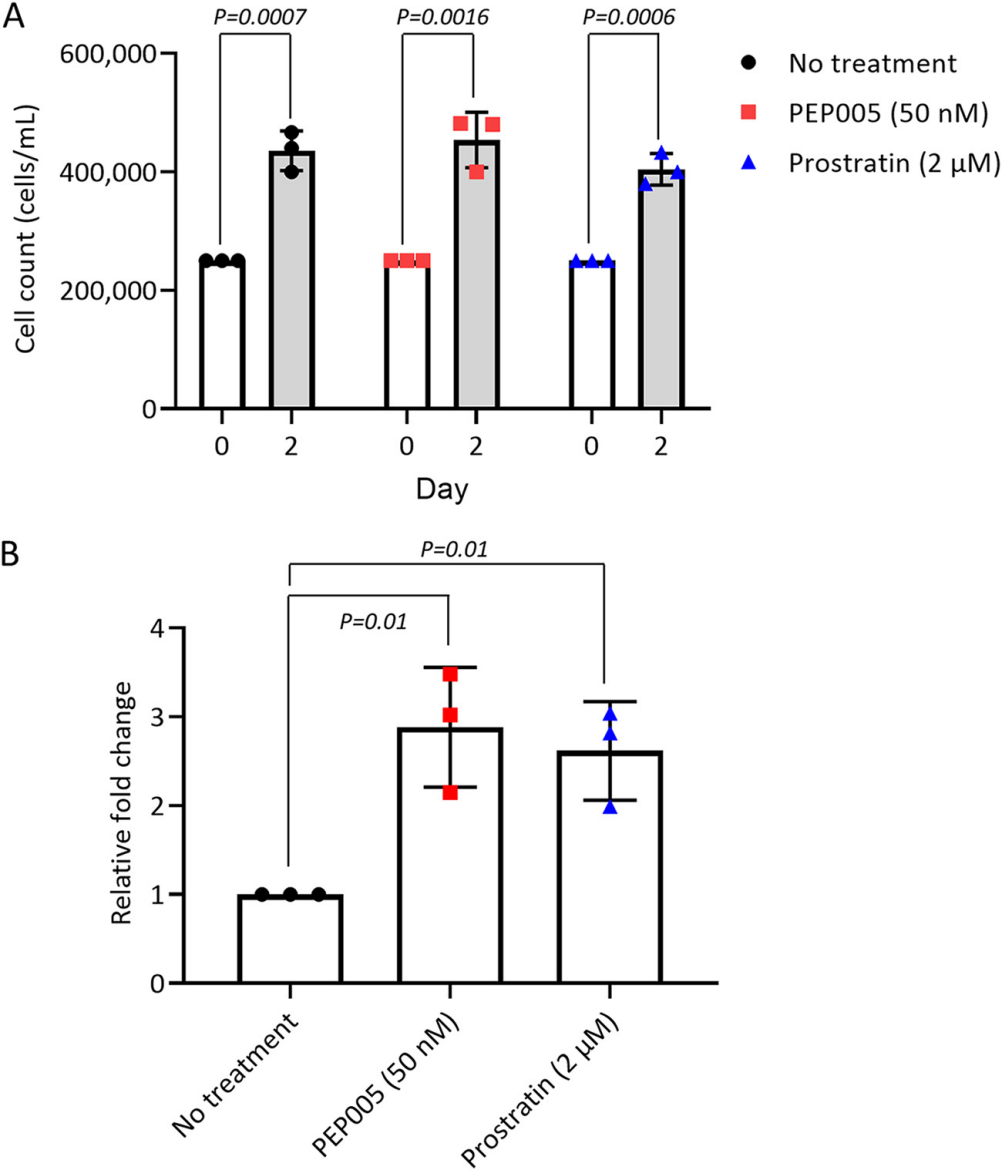
Enhancement of dengue virus (DENV) defective interfering particle (DIP) production in HEK-DI-290-ORF cells by the PKC/NF-κB activating agents. HEK-DI-290-ORF cells were treated with PEP005 (50 nM) or prostratin (2 μM) for 2 days. (A) Cell density in no-treatment control, PEP005-treated, and prostratin-treated groups on day 0 and day 2. (B) Levels of DI-290 RNA in culture supernatants were measured by reverse transcription-quantitative PCR (RT-qPCR). Data are presented as fold change relative to the no-treatment control group. Data are expressed as the mean ± standard deviation (SD) from three experiments. *P* values were calculated using a two-tailed Student’s *t* test.

HEK-DI-290-ORF cells were grown in a spinner flask bioreactor to produce DENV DIPs in SFM. We used ingenol-3-angelate (also called PEP005) and prostratin, which activate the protein kinase C (PKC)/nuclear factor kappa B (NF-κB) signaling pathway (21, 22) and at low concentrations are minimally toxic to cells (23), to test whether their addition improved DIP/DI-290 RNA levels in culture supernatant. The bioreactors were seeded with HEK 293-DI-290-ORF cells in SFM containing 50 nM PEP005 or 2 μM prostratin. The densities of treated and untreated cells in SFM after 48 h were unaffected by either compound (Fig. 1A). However, the amount of DI-290 RNA in pelleted DIPs increased by 2.9-fold for PEP005 and 2.6-fold for prostratin (Fig. 1B). For DIP production, 50 nM PEP005 was added to SFM.

### Purification of DENV DIPs.

**FIG 2:**
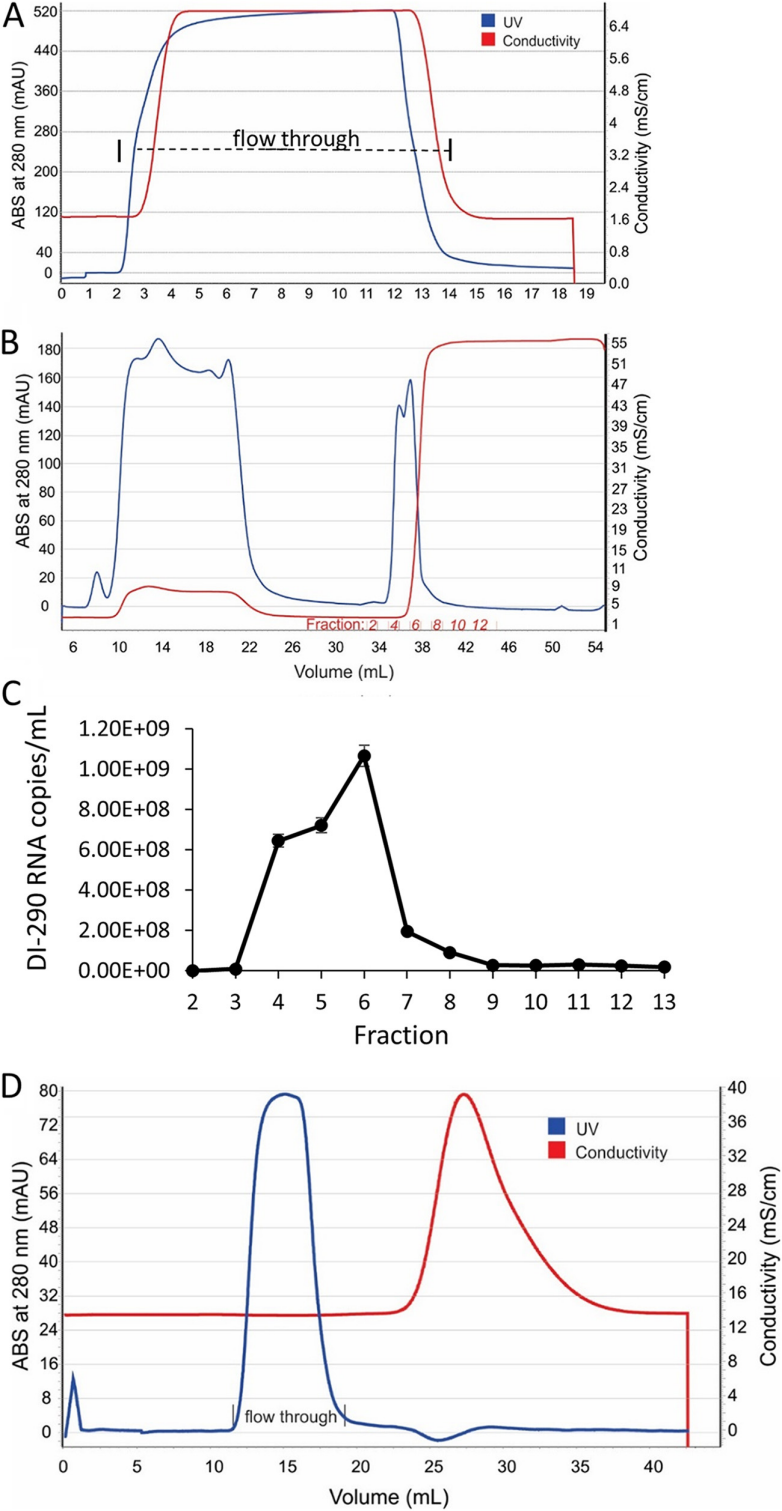
Three-step chromatographic purification of DENV DIPs. (A) Chromatogram of the multimodal Capto Core 400 (CC400) column step of DENV DIP purification. The flowthrough fraction collected is indicated. (B) Chromatogram of the capture step for DENV DIPs using ceramic hydroxyapatite (CHT) medium, which was washed with 10 mM sodium phosphate buffer (pH 7.2) and eluted with an isocratic gradient using 0.6 M sodium phosphate buffer (pH 7.2). The fractions collected in the elute phase are indicated. (C) Samples from each fraction underwent ultracentrifugation. The pelleted material was collected and assayed for DI-290 RNA by RT-qPCR. The mean numbers of DI-290 RNA copies/mL measured in each fraction in triplicate assays are shown. Independent experiments were conducted where DI-290 RNA was always associated with the milli-absorbance unit (mAU) peak absorbance (ABS) at 280 nm. (D) The CHT elution peak as applied to a Cytiva G-25 desalting column equilibrated in 1× PBS and operated in flowthrough mode.

A four-step procedure was used to purify DIPs from clarified HEK 293-DI-290-ORF cell culture supernatant. At each step, the recovery of DIPs was measured by ultracentrifugation of samples and analysis of DI-290 RNA levels in pelleted material. Only DI-290 RNA associated with high mass complexes such as DIPs will pellet, and the level of this RNA was measured by reverse transcription-quantitative PCR (RT-qPCR). The first purification step was precipitation of DIPs using polyethylene glycol (PEG) 8000 buffer as previously described (24). After centrifugation, the pelleted materials were dissolved in sodium phosphate buffer (pH 7.2). RT-qPCR analysis of the PEG supernatant showed that approximately 50% of the DI-290 RNA measured in culture supernatant was recovered in the PEG-precipitated material (Table 1). The solubilized material was applied to a 2 mL Capto Core 400 (CC400) mixed-modal column equilibrated in sodium phosphate buffer (pH 7.2) that was operated in flowthrough mode. CC400 columns use anion exchange porous beads which allow negatively charged molecules with a mass of >400,000 Da to enter and bind to the matrix. DIPs and other macromolecules which exceed the bead exclusion limit or fail to bind the matrix are collected in the flowthrough fraction. We observed in repeated purification runs that more than 80% of DI-290 RNA measured in PEG-precipitated material was recovered in the CC400 flowthrough fraction (Fig. 2A, Table 1).

**TABLE 1:** Purification of DENV DIPs^*a*^

Purification step	Protein		DIP		DI-290 RNA	
	Conc. (μg/mL)	Total (μg)	Conc. (RNA copies/mL)	Total RNA copies	% Recovery	Copies/μg of total protein
DIP sup.	1,754	2.63E + 6	5.36E + 07	8.04E + 10	NA	3.06E + 04
PEG prec.	575	8.6E + 3	2.88E + 09	4.31E + 10	54	5.01E + 06
CC400 column	397	5.96E + 3	2.33E + 09	3.50E + 10	44	5.87E + 06
CHT column	55	825	1.51E + 09	2.26E + 10	28	2.74E + 07
Desalting column	38	770	1.22E + 09	2.28E + 10	28	4.0E + 07

a DENV, dengue virus; DIP, defective interfering particle; sup., supernatant; NA, not applicable; PEG, polyethylene glycol; prec., precipitate; CC400, Capto Core 400; CHT, ceramic hydroxyapatite.

CHT has been used previously to purify viruses, including DENV and IAV, from culture supernatant (25). A CHT column operated in elute mode was used to purify DIPs. The CC400 flowthrough fraction was applied to a 10-mL CHT column (Fig. 2B). DIP/DI-290 RNA complexes were retained on the column while unbound protein eluted in the flowthrough fraction. Only low levels of DI-290 RNA, approximately 1% of total DI-290 RNA in the CC400 flowthrough fraction, were measured in the CHT unbound fraction by RT-qPCR. The column was washed with the same buffer, and DIPs/DI-290 RNA complexes were eluted with a 0.6 M sodium phosphate buffer (pH 7.2) isocratic gradient, which was collected in 1-mL fractions. Ultracentrifugation was used to pellet high-molecular weight complexes from a sample of each fraction, and levels of DI-290 RNA copies were measured by RT-qPCR. DI-290 RNA coeluted in the fractions which had peak absorbance at 280 nm (Fig. 2C). The CHT peak fractions were pooled and passed over a G-25 desalting column equilibrated with 1× PBS (phosphate-buffered saline), and the DIP/DI-290 RNA complexes were collected in the flowthrough fraction (Fig. 2D). Next, hydrolyzed gelatin and sorbitol were added to the purified DIPs (26, 27). We confirmed that DENV DIPs stored at 4°C retain antiviral activity for at least 3 weeks (Fig. S1).

Table 1 summaries an outcome from a typical DENV DIP purification scheme with respect to DIP recovery and final protein concentration of purified DIPs. The recovery of DIPs in 1× PBS from the G-25 desalting column was approximately 53% compared to the total amount of DIPs measured in the PEG-precipitated material. DIP concentration in 1× PBS increased approximately 30-fold. However, the concentration of DIPs in 1× PBS relative to the total protein in the preparation increased approximately 1,000-fold.

### DENV DIPs are a small virus-like particle.

**FIG 3:**
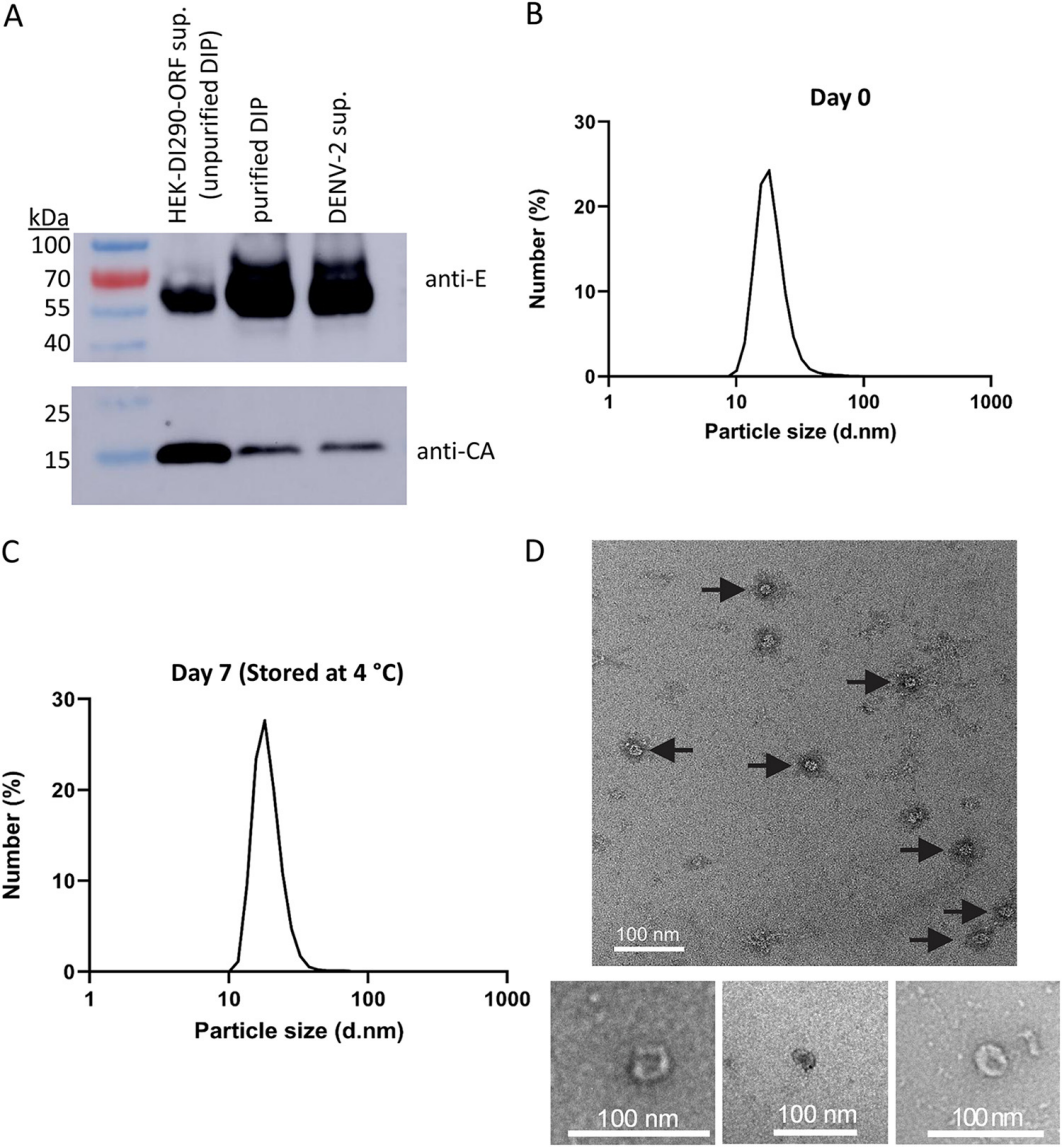
Size and morphology of purified DENV DIP particles. (A) Samples containing unpurified DENV DIPs, purified DIPs, or infectious DENV-2 underwent ultracentrifugation. The pelleted material was solubilized and used for Western blot analysis using anti-DENV antibodies directed to the viral envelope (E) or capsid (CA) proteins. (B) Dynamic light scattering (DLS) analysis of purified DENV DIP particle size distribution or (C) DLS of the same DENV DIP preparation after an additional 7 days of storage at 4°C. (D) Transmission electron microscopy (TEM) images (scale bar = 100 nM) of purified DENV DIPs.

Dengue virions are spherical, approximately 40 to 60 nm in diameter and composed of a nucleocapsid made of viral RNA and capsid (C) protein. The nucleocapsid is surrounded by a lipid membrane bilayer embedded with envelope (E) and membrane (M) proteins. A Western blot of unpurified DIPs, purified DIPs, and infectious DENV-2 confirmed that both E and C proteins were present and that the relative ratios of E to C in purified DIP and DENV-2 were similar (Fig. 3A). Dynamic light scattering (DLS) was performed after purification to estimate the size of DENV DIPs. DLS of purified DIPs indicated an average size of 19 nm (Fig. 3B), with a range of 13 to 40 nm. The particle size distribution was unchanged after the particles were measured again after incubation in 1× PBS for an additional 1 week at 4°C (Fig. 3C). DENV DIPs were examined by transmission electron microscopy (TEM) using negative staining. Fig. 3D shows TEM images using purified DENV DIP and confirmed the successful production and purification of virus-like particles, most of which were approximately 19 nm in diameter.

### DENV DIPs strongly inhibit infectious DENV-2 and induce interferon responses in Huh7 cells.

**FIG 4:**
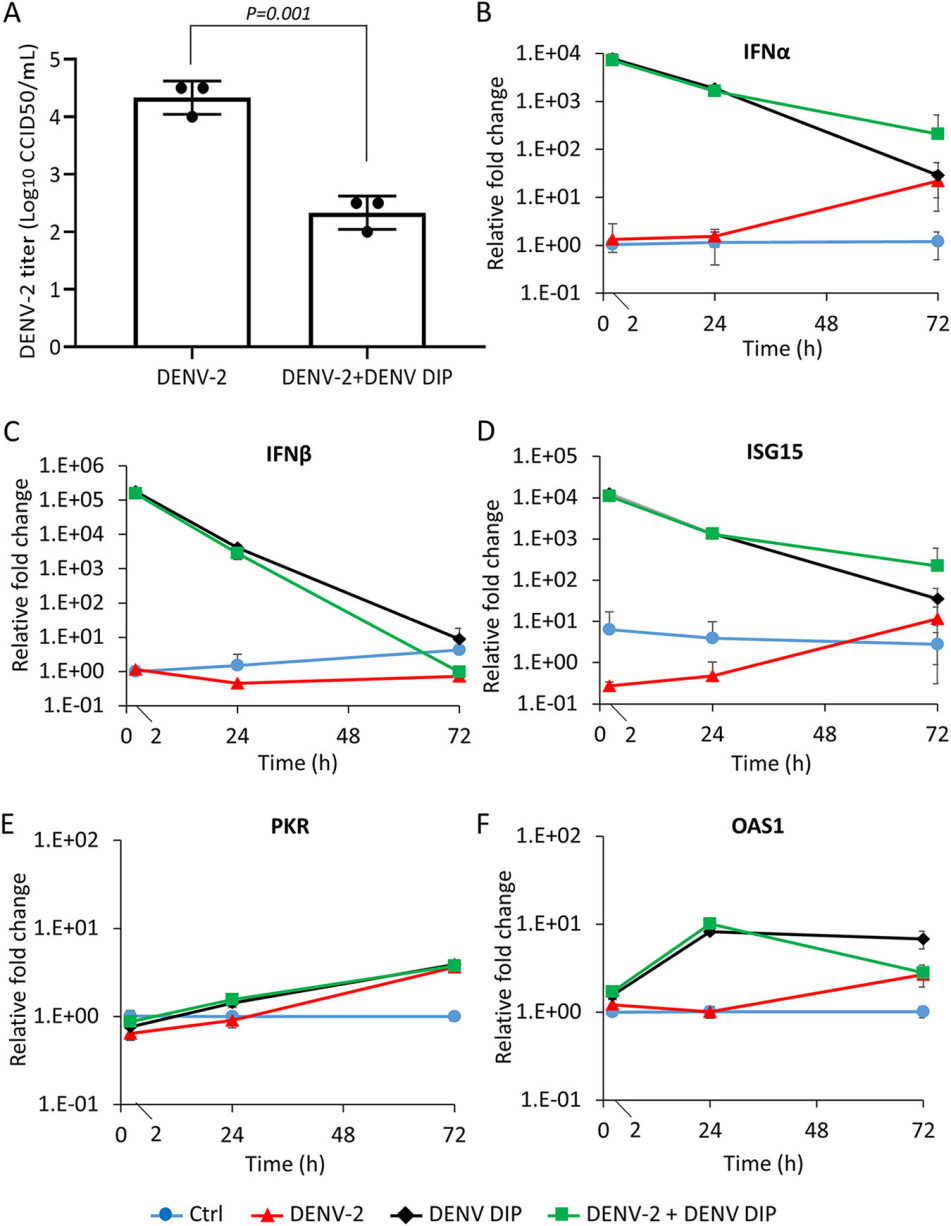
DENV DIPs stimulate host innate immune responses. (A) Huh7 cells were incubated with DENV-2 (MOI [multiplicity of infection] = 0.01 CCID_50_ [50% cell culture infective dose]/cell) alone, or DENV-2 mixed with DENV DIP (equivalent to 1,000 DI-290 RNA copies/cell) for 15 h, and then the culture medium was replaced. Culture supernatants were collected at 72 h postinfection (hpi). Viral titers in culture supernatants were measured by a CCID_50_ assay. (B to F) To examine host innate immune response, Huh7 cells were treated with DENV-2 (MOI = 0.01 CCID_50_/cell, red line), DENV DIP (equivalent to 1,000 DI-290 RNA copies/cell, black line), or DENV-2 mixed with DENV DIP (green line) for 2, 24, and 72 h. Total RNA was extracted from the cells and the levels of interferon (IFN)-α, IFN-β, ISG15, protein kinase R (PKR), and 2′–5′ oligoadenylate synthetase 1 (OAS1) mRNA were quantified by RT-qPCR. The fold change relative to the untreated control cells was calculated (Ctrl, blue line). Data are shown as means ± SD from three replicate experiments. The *P* values were calculated using a two-tailed Student’s *t* test.

We previously showed that DENV DIPs inhibited DENV-2 replication *in vitro* (20). Here, we confirmed that purified DENV DIPs inhibited infectious DENV-2 replication and reduced viral titers in supernatant from in Huh7 cell culture compared to that in untreated infected cells (Fig. 4A). Recently, it was shown that *in vitro*-transcribed DI-290 RNA transfected into Huh7 cells stimulated a host cell interferon-mediated antiviral response (28). To assess whether purified DIPs stimulated cellular innate immune responses, DENV-2 alone, DENV DIPs alone, or DENV-2 mixed with DIPs (DENV-2+DENV DIPs) were added to Huh7 cells. After 2, 24, and 72 h post-addition, the levels of host mRNA for IFN-α, IFN-β, IFN-stimulated gene 15 (ISG15), 2′–5′ oligoadenylate synthetase 1 (OAS1), and protein kinase R (PKR) were measured in cells. Interestingly, 2 h after DIPs or DENV-2+DENV DIPs were added to cells, IFN-α and IFN-β mRNA levels were strongly upregulated by ~7,000- (Fig. 4B) and ~150,000 (Fig. 4C) fold, respectively. The levels of IFN-α and IFN-β mRNA decreased after 24 and 72 h. As expected, treating cells with poly(I:C), a double-stranded RNA analogue, stimulates Toll-like receptor 3 (TLR3) signaling and upregulates IFN-α and IFN-β mRNAs. Interferon responses resulting from poly(I:C) exposure were inhibited by pre-treating poly(I:C) with benzonase nuclease, which degrades poly(I:C) (29). However, benzonase nuclease treatment of DIPs had no effect on the upregulation of IFN-α and IFN-β mRNAs, suggesting that DIPs stimulated interferon responses independently of TLR3 signaling (Fig. S2), and that DIPs protected DI-290 RNA from degradation by benzonase nuclease. In addition, we observed that the expression of ISG15, a strongly upregulated ISG, was increased by ~10,000-fold 2 h after the addition of DENV DIPs to Huh7 cells (Fig. 4D). Likewise, DENV DIPs alone and DENV-2+DENV DIPs also stimulated PKR (Fig. 4E) and OAS1 (Fig. 4F) antiviral gene expression. Overall, these data indicate that DENV DIPs strongly induce interferon responses to activate an antiviral state in Huh7 cells.

### Inhibition of ZIKV, YFV, RSV, and SARS-CoV-2 by DENV DIPs *in vitro*.

**FIG 5:**
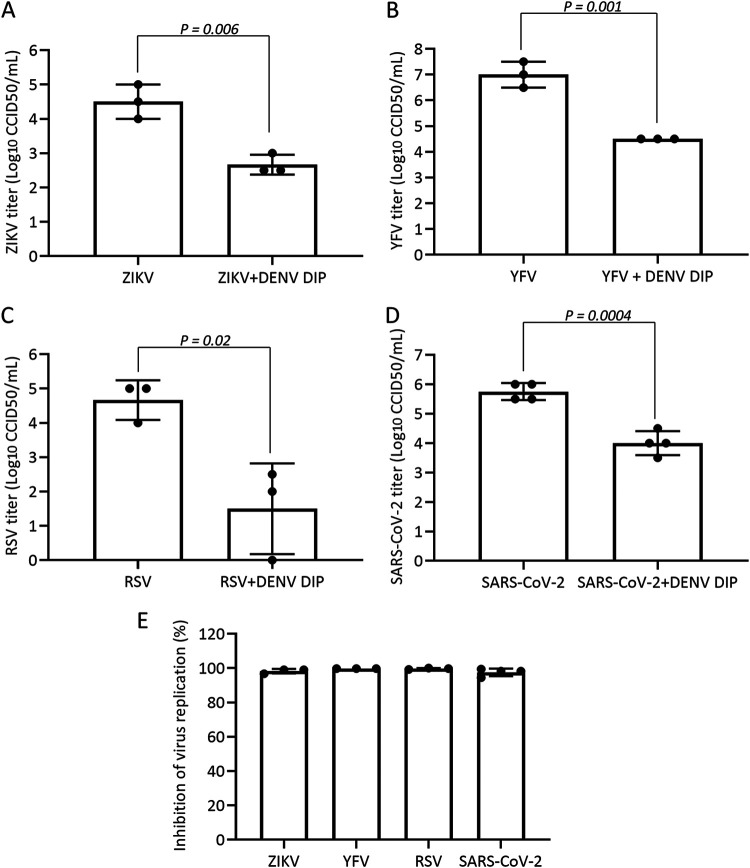
DENV DIP inhibits Zika virus (ZIKV), yellow fever virus (YFV), respiratory syncytial virus (RSV), and severe acute respiratory syndrome coronavirus 2 (SARS-CoV-2). Huh7 or HEp-2 cells were incubated with ZIKV (MOI = 0.01 CCID_50_/cell), ZIKV mixed with DENV DIP (equivalent to 1,000 DI-290 RNA copies/cell), YFV (MOI = 0.01 CCID_50_/cell), YFV mixed with DENV DIP, RSV (MOI = 0.5 CCID_50_/cell), RSV mixed with DENV DIP, SARS-CoV-2 (MOI = 0.01 CCID_50_/cell), or SARS-CoV-2 mixed with DENV DIP for 15 h, after which the culture medium was replaced. Culture supernatants were collected at 72 hpi. Viral titers in culture supernatant samples were measured by CCID_50_ assay (A to D). (E) Level of virus replication inhibition by DIPs. Data are shown as means ± SD from at least three replicate experiments. *P* values were calculated using a two-tailed Student’s *t* test.

DIPs derived from IAV could inhibit replication of SARS-CoV-2 presumably through immunostimulatory effects on cells (12). Here, we assessed whether DENV DIPs could inhibit the replication of the heterologous flaviviruses ZIKV and YFV-17D, or RSV strain A2 and SARS-CoV-2 (omicron BA.1 variant), which are unrelated viruses with negative-sense and positive-sense RNA genomes, respectively. Huh7 or HEp-2 cells were infected with each virus mixed with DENV DIPs containing the equivalent of 1,000 DI-290 RNA copies/cell. After overnight incubation, the culture medium was replaced, and samples of supernatant were collected and assayed for viral titers at 72 h postinfection (hpi). ZIKV and YFV viral titers were decreased by ~2- and ~3 log, respectively, in cells treated with DENV DIPs (Fig. 5A and B). We also discovered that DENV DIPs inhibited RSV and SARS-CoV-2 by ~1,500- and ~60-fold, respectively (Fig. 5C and D). These data demonstrate that DIPs can inhibit other members of *Flavivirus* and other RNA viruses such as RSV and SARS-CoV-2 by at least 98% in cells, most likely by inducing innate immune responses (Fig. 4 and 5).

## DISCUSSION

In this study, a cell culture-based system was used to synthetically produce clonal DENV DIPs containing a 290-nt long DI RNA which were used to treat cells infected with five different RNA viruses. Here, we show that cells infected with DENV, ZIKV, YFV, RSV, or SARS-CoV-2 and treated with DENV DIPs had virus replication reduced by 98% or more compared to that in untreated cells (Fig. 5E). DIP-treated cells rapidly induced interferon-mediated antiviral responses. For this reason, it is possible that DENV DIPs have broad-acting antiviral activity against a large number of RNA viruses; this possibility is under investigation.

Exactly how DENV DIPs stimulate cellular interferon responses will require further investigation, but it most likely involves retinoic acid-inducible gene I (RIG-I) (30). RIG-I is a cytosolic pattern recognition receptor triggered by small double-stranded viral RNA and activated during DENV infections (31). It has been demonstrated that synthetic *in vitro*-transcribed RNA nearly identical to 290-DI RNA transfected into Huh7 cells strongly upregulates interferon responses (28). However, the same RNA transfected into Huh7.5 cells, which have a defective RIG-I gene, exhibited attenuated interferon responses. In addition, the DENV-2 5′ UTR alone, which is contained in DI-290 RNA, was shown to stimulate cellular interferon response by a RIG-I-dependent mechanism (32). We propose that MAD5 or TLR3 are less likely or unlikely to mediate interferon response by DENV DIPs. While we cannot rule out MAD5 activation, which is triggered by dengue infection, MAD5 recognizes long double-stranded RNA (33). TRL-3 also detects long double-stranded viral RNA as well as via poly(I:C) (34). The fact that nuclease prevented cellular interferon stimulation by poly(I:C) but not DENV DIPs (Fig. S2) indicates that the DI-290 RNA was protected from nuclease attack by DIP packaging. It is likely that DENV DIP entry and the release of DI-290 into the cytosol leads to detection by RIG-I and interferon induction.

All flaviviruses encapsidate a positive-sense RNA genome of about 11 kB and have a 5′ UTR and 3′ UTR, which have a conserved RNA secondary structure that is essential for viral RNA replication (35). DI-290 RNA contains the entire 5′ and 3′ UTRs. Although the sequence identity of the 5′ UTR between different *Flavivirus* species is low, a region of the 5′ UTR called stem-loop A (SLA) shares a similar Y-shape RNA structure that functions as a promoter for viral RNA synthesis via the viral NS5 RNA polymerase. With respect to DENV-2 infection, we previously showed that DI-290 replicates in cells using the DENV-2 RNA polymerase replication complex (20), suggesting that competition for viral and cellular resources by DI-290 RNA contributes to anti DENV-2 activity. Whether DI-290 inhibits both ZIKV and YFV by competitive inhibition is unknown and requires further study. Very few studies have investigated interactions between NS5 and SLA from different *Flavivirus* species. In one study, a chimeric West Nile virus (WNV) containing the DENV-2 SLA in its 5′ UTR replicated as efficiently as wild-type WNV, indicating that DI-290 RNA can interact (36), but whether the DENV-2 SLA in DI-290 RNA competes for binding to ZIKV or YFV NS5 is unknown.

Our study showed that DENV DIP production is scalable. Here, stir flask bioreactors were used to produce DENV DIPs in SFM. DENV DIPs were purified and concentrated to a concentration of >10^9^ DI-290 RNA copies/mL (Table 1). DIP production by HEK-DI-290-ORF cells in bioreactors was remarkably stable after months of DIP production by HEK-DI-290-ORF cells. This feature of HEK-DI-290-ORF cells should support future large-scale, commercial production of DENV DIPs. As we previously reported (20) and in this study, DIPs package DI-290 RNA, which was used to measure DIP concentrations in culture supernatant. HEK-DI-290-ORF cells typically produce approximately 2 × 10^6^ to 2 × 10^7^ DI-290 RNA copies/mL. Here, we tested the PKC activators PEP005 and prostratin for the ability to stimulate HEK-DI-290-ORF cells and increase DIP production. PKC comprises a family of serine/threonine kinases with at least 12 isoenzymes that are effectors of diacylglycerol (37). PKC isoforms play important roles in many different cellular events, including cellular proliferation, cell cycle progression, differentiation, apoptosis, and tumor angiogenesis (38). Prostratin and PEP005 can induce the translocation of both conventional and novel PKC isoforms, and their prolonged exposure to cells typically leads to reduced cellular levels of various PKC isoforms in a concentration-dependent manner (39–41). Both compounds are also HIV-1 latency reversal agents that are believed to stimulate virus gene expression in cells through activation of NF-κB transcription factor and the positive transcription elongation factor to improve RNA transcription by RNA polymerase II. Because activators of PKC can inhibit cell proliferation and induce apoptosis, we tested the concentrations of each compound reported to stimulate HIV-1 gene expression but not affect cell proliferation. While short term proliferation of HEK-DI-290-ORF cells was unaffected by either compound, both compounds significantly increased DIP production (Fig. 1B). In repeated cycles of PEP005 treatment in HEK-DI-290-ORF cells, no detrimental effects on cell proliferation or DIP production were observed (Fig. 1A). It is possible that PEP005 could be useful to stimulate VLP production in other cell culture systems.

A four-step column chromatography process was used to purify DIPs from SFM. The final DIP preparation removed ~99.97% of protein measured in the original SFM DIP supernatant and retained ~28% of DIP-associated DI-290 RNA (Table 1). The purified DENV2-based DIPs and infectious DENV had very similar elution profiles in sodium phosphate buffers, and Western blots showed that the apparent ratios of E and C proteins were also similar (Fig. 3A). We speculate that CHT purification could select for DIPs with higher E levels because Western blots suggest that the E to C protein ratio in DIPs from SFM is lower than that in purified DIPs.

Testing showed that purified DIPs retained antiviral activity for 3 weeks when stored at 4°C in a sodium phosphate buffer containing the stabilizers gelatin and sorbitol, which are sometime used for storage of attenuated viral vaccines (27). It was observed that VLPs stored in PBS retain a membrane-bound VLP structure, which was visualized by TEM (Fig. 3). DIPs were spherical with an average diameter of 19 nm (range: 10 to 40 nm), which is less than half the size of infectious DENV with an average diameter of 40 to 60 nm. The core structure of DENV is usually about 25 to 30 nm, but DIPs had a very diffuse-looking core, possibly due to the small DI-290 RNA which contains only about 2.6% of the size of DENV viral genome. VLPs for ZIKA and YF have been reported which are produced without a viral genome, spherical, and about the same diameter as the infectious virus (42). So far, the number of DI-290 RNA copies packaged in a DIP has not been determined, but it seems likely that each DIP encapsidates multiple copies of DI-290 RNA. Knowledge of how DENV selects and packages viral RNA is limited. The highly basic viral capsid protein binds to viral genomic RNA that is specifically packaged in nascent virions (43), but so far no RNA packaging signal has been identified. It has been suggested that the 3′ UTR plays a role because viral nonstructural proteins are capable of packaging subgenomic replicons (43). HEK-DI-290-ORF cells produce all DENV structural and nonstructural proteins which permit replication of DI-290 RNA. It is tempting to speculate that DI-290 RNA replication and packaging are coordinated in HEK-DI-290-ORF cells. It has been hypothesized that genome length and virion volume conform to a simple allometric scaling law (44). It has been suggested that viral genome sizes are constrained by what can be physically packaged within a single virion. The data here suggest that encapsidation of small DI-290 RNA(s) led to small DENV VLPs, a phenomena observed in viruses in which virion copy number or genome size regulates virion biophysical parameters such as virion size and particle density (45, 46). Previously, we reported that DENV DIPs are less dense than infectious DENV (20).

The purified DENV DIPs described in this report will enable future *in vivo* preclinical toxicity investigation and antiviral testing. For example, knockout mice lacking the IFN-α/β receptors can be used to study DENV infection (47). DENV DIPs activate type 1 interferon responses in Huh7 cells (Fig. 4), so it would be interesting to test DENV DIP inhibition of DENV replication in an animal system lacking a functional type 1 interferon response. Previously, we showed that DENV DIPs inhibited DENV replications in interferon-deficient Vero cells, suggesting that DIPs inhibit virus replication by competing for viral and cellular resources (20). The antiviral activity of DENV DIPs might also be tested wild-type mice infected with SARS-CoV-2 or other respiratory viruses. Wild-type mice treated with poliovirus-derived DIPs or DI RNA exhibited strongly stimulated innate immune responses and improved survival after a potentially lethal viral infection (48). Animal models may also be used to determine whether DENV DIPs induce humoral immune responses such as anti-DENV antibodies. Because antibody-dependent enhancement (ADE) is a concern for people previously infected with DENV (49), the impact of DENV DIP treatment on ADE risk will need to be investigated in future studies.

Overall, the work here presents a pathway for DIP production that is adaptable to Good Manufacturing Practice and preclinical testing, and, with preliminary successes, should be suitable for evaluation in subjects. For example, DIPs could be tested for the ability to inhibit virus replication in subjects immunized with a live attenuated dengue vaccine such as TV003, developed at the Laboratory of Infectious Diseases at the National Institutes of Health (USA) (50). However, type 1 interferons induced by DIPs could assist in other health areas, such as in targeted and personalized anti-cancer treatments (51).

## MATERIALS AND METHODS

### Cell culture.

Vero E6 (Vero), HEp-2, and Huh7 cells were maintained in Dulbecco’s Modified Eagle Medium (DMEM; Thermo Fisher Scientific) supplemented with 10% (vol/vol) fetal bovine serum FBS; (Thermo Fisher Scientific) and 1% (vol/vol) penicillin-streptomycin (Thermo Fisher Scientific). Suspension-adapted HEK-DI-290-ORF cells were cultured in EX-CELL 293 Serum-Free Medium (Sigma-Aldrich) supplemented with 1% (vol/vol) penicillin-streptomycin, 1% (vol/vol) GlutaMAX-I (Thermo Fisher Scientific), and 50 nM PEP005 during DENV DIP production. All cells were incubated at 37°C in a humidified 5% CO_2_ atmosphere. C6/36 cells were grown in DMEM supplemented with 10% (vol/vol) FBS and 1% (vol/vol) penicillin-streptomycin at 28°C in a humidified 5% CO_2_ atmosphere.

### Analysis of DENV DIP antiviral activity.

Huh7 or HEp-2 cells were seeded in 48-well plates with 15,000 cells/well. The following day, the Huh7 cells were washed with 1× PBS and incubated with purified DENV DIPs (equivalent to 1,000 DI-290 RNA copies per cell) and either prototype strain of DENV-2 (20), ZIKV strain MR766 (52), live attenuated YFV 17D vaccine strain (53), or SARS-CoV-2 omicron variant (BA.1, GenBank ID: ON819429.1) (54) at minimum inhibitory concentrations (MOIs) of 0.01 CCID_50_ (50% cell culture infective dose)/cell. The HEp-2 cells were cotreated with DENV DIPs (equivalent to 1,000 DI-290 RNA copies per cell) and the RSV A2 strain at an MOI of 0.5 CCID_50_/cell. After 15 h, the culture medium was replaced. At 3 days postinfection, 200 μL of culture supernatant was collected and clarified by centrifugation at 2,000 × *g* for 5 min. The concentrations of vRNA in the culture supernatants were measured by RT-qPCR and the viral titers were determined by CCID_50_ assay.

### Quantification of supernatant vRNAs.

The clarified supernatants were pelleted by ultracentrifugation at 100,000 × *g* for 1 h at 4°C. The pelleted materials were resuspended in 20 μL H_2_O containing 0.5% Triton X-100 (Sigma-Aldrich) and 20 U of RNasin RNase inhibitor (Promega). Two μL of resuspended samples was used to quantify DI-290 RNA and DENV-2 NS5 RNA regions using a Luna Universal One-Step RT-qPCR kit (New England Biolabs) in a 10-μL reaction volume. The thermal cycling conditions were adapted from a protocol used to detect SARS-CoV-2 without RNA extraction: 15 min at 55°C; 2 min at 95°C; and 40 cycles of 5 sec at 95°C, 45 sec at 55°C (data acquisition), and 15 sec at 72°C (55). The primer sequences are available on request.

### CCID_50_ assay.

Virus titers in culture supernatants were determined by CCID_50_ assay as described previously (56). Briefly, the supernatants were titrated in duplicate in 10-fold serial dilutions on C6/36 cells. After 5 days, 50-μL volumes of supernatants were transferred in parallel onto 96-well plates containing Vero cells. After another 5 days of culture, virus-positive wells were determined by cytopathic effects. To determine RSV and SARS-CoV-2 titers, the culture supernatants were titrated in duplicate in 10-fold serial dilutions on Vero cells in 96-well plates. RSV- or SARS-CoV-2-positive wells were examined by cytopathic effects after 6 days of culture (57). The titers were then calculated by the Spearman and Kärber algorithm (58).

### Gene expression analysis.

Huh7 cells were seeded at a density of 50,000 per well in a 48-well plate. The next day, the cells were infected with either DENV-2 at an MOI of 0.01 CCID_50_/cell, purified DENV DIPs (equivalent to 1,000 DI-290 RNA copies per cell), or DENV-2 mixed with purified DENV DIPs. For poly(I:C) stimulation, the cells were treated with poly(I:C) (Sigma-Aldrich) at a concentration of 5 μg/mL. Total RNA from cells was extracted after 2, 24, and 72 h postinfection using an RNeasy kit (Qiagen), and the mRNA expression levels of GAPDH (glyceraldehyde-3-phosphate dehydrogenase), IFN-α, IFN-β, OAS1, PKR, and ISG15 were determined using a Luna Universal One-Step RT-qPCR kit according to the manufacturers’ instructions. The primer sequences are available upon request. The data were normalized to RPL13a as the reference gene and presented as fold change relative to untreated, uninfected control cells using the threshold cycle (ΔΔ*CT*) method.

### Western blot analysis.

Culture supernatants from HEK-DI-290-ORF cells or DENV-2 infected HEK 293T cells, or purified DENV DIP supernatant, were ultracentrifuged at 100,000 × *g* for 1 h at 4°C. The pelleted materials were boiled in SDS-PAGE sample buffer (125 mM Tris-HCl [pH 6.8], 4% [vol/vol] SDS, 20% [vol/vol] glycerol, 0.004% [wt/vol] bromophenol blue) and separated by 4 to 20% precast SDS-PAGE gels (Bio-Rad). Gels were electroblotted onto a polyvinylidene fluoride (PVDF) membrane (Pall) using a semi-dry transfer system (Bio-Rad). DENV-2 E and CA proteins were detected with a rabbit anti-DENV E polyclonal antibody (GeneTex) and a rabbit anti-DENV CA polyclonal antibody (Novus Biologicals), respectively. Primary antibodies were detected with anti-rabbit IgG horseradish peroxidase (HRP)-linked antibodies (Cell Signaling Technology).

### Quantitation of total protein.

Total protein content in samples from each purification step was measured by Pierce BCA Protein assay kit (Thermo Fisher Scientific) following the manufacturer’s protocol.

### DENV DIP production, precipitation, and chromatographic purification.

HEK-DI-290-ORF cells were seeded at 2.5 × 10^5^ cells/mL and grown in a ProCulture glass spinner flask (Corning) using a magnetic stir plate (Thermo Fisher Scientific) set at 65 rpm. After 48 h, the culture supernatant containing DENV DIPs was collected, filtered through a 0.22-μm filter (Merck Millipore), and stored at 4°C. The clarified culture supernatant was mixed with PEG 8000 to a final concentration of 10% and 0.25 M NaCl (pH 7.6). The mixture was stirred overnight at 4°C. The PEG precipitate was pelleted at 8,000 × *g* for 2 h at 4°C, and the pelleted material was resuspended in 10 mM Tris (pH 7.2). The suspension was passed through a 0.22-μm filter before chromatographic purification of DENV DIPs.

All chromatographic steps were performed using an ÄKTA Start protein purification system controlled by the software Unicorn Start 1.1 (Cytiva). The chromatography was performed in three steps. The first step used two 1-mL HiTrap CC400 columns connected in series and operated in flowthrough mode (Cytiva). The clarified PEG solution, about 10 to 15 mL, was applied to the column at 2 mL/min and the flowthrough fraction was collected. Next, two 5-mL Bio-Scale Mini CHT type II, 40-μM cartridges connected in series (Bio-Rad, cat no. 7324332) were pre-equilibrated with 10 mM sodium phosphate buffer (pH 7.2). CHT is a spherical macroporous hydroxyapatite bead composed of calcium and phosphate salts. CHT is both the matrix and the ligand. The flowthrough from the CC400 column was applied to the CHT column and washed with 30 mL of 10 mM sodium phosphate buffer (pH 7.2) each at 2 mL/min. Macromolecules bound to the CHT column were eluted using 30 mL of 600 mM sodium phosphate buffer in an isocratic gradient at 2 mL/min. When the UV absorbance at 280 nm (ABS at 280 nm) of the eluate reached 15 mAU, the peak fraction was collected and stopped when the ABS at 280 nm fell to 15 mAU. Typically, the volume of the peak fraction ranged from 8 to 11 mL. The final step was a desalting column (Cytiva, cat no. 29-0486-84) made of Sephadex G-25 Superfine, where 5 mL of medium was used for each 1.5 mL of CHT column eluate operated in flowthrough mode. The desalting column was pre-equilibrated in DIP storage buffer composed of 1× PBS, 1.8% gelatin (Sigma-Aldrich, cat no. G0262), and 5% sorbitol (Sigma-Aldrich, cat no. 85529). The CHT peak fraction was applied to the desalting column by syringe, and the sample was eluted in equilibration buffer collecting the flow through fraction.

### Dynamic light scattering.

The particle size and stability of DENV DIPs in 1× PBS were measured using the DLS approach on a Nano Zetasizer (Malvern). The stability of DENV DIPs was determined by measuring the particle size distribution of DIPs in 1× PBS stored for 4 and 11 days at 4°C.

### Transmission electron microscopy.

Microscopy images were obtained by Lou Brillault at the University of Queensland Centre for Microscopy and Microanalysis. The DENV DIP sample on the TEM grid was prepared by negative staining using uranyl acetate. TEM images of DENV DIPs were captured on a Tecnai F30 at 100 kV with magnification at ×59,000 and ×155,000.

### Statistics and reproducibility.

Statistical analysis was performed with a two-tailed Student’s *t* test from at least three independent experiments or measurements. Statistical significance was set at *P* < 0.05.
