# Supplementary material for: Defective Interfering Particles with Broad-Acting Antiviral Activity for Dengue, Zika, Yellow Fever, Respiratory Syncytial and SARS-CoV-2 Virus Infection
Source: Microbiol Spectr. 2022 Nov 29;10(6):e03949-22. doi: 10.1128/spectrum.03949-22 (PMC9769664; doi:10.1128/spectrum.03949-22)
Supplement: Supplemental file 1 — Supplemental material. Download spectrum.03949-22-s0001.pdf, PDF file, 0.5 MB [file spectrum.03949-22-s0001.pdf]

Supplementary materials for:

Defective interfering particles with broad-acting antiviral activity for dengue, Zika, yellow fever, respiratory syncytial and SARS-CoV-2 virus infection.

Min-Hsuan Lin[1], Dongsheng Li[1], Bing Tang[1], Li Li[2], Andreas Suhrbier[1], and David Harrich[1]*

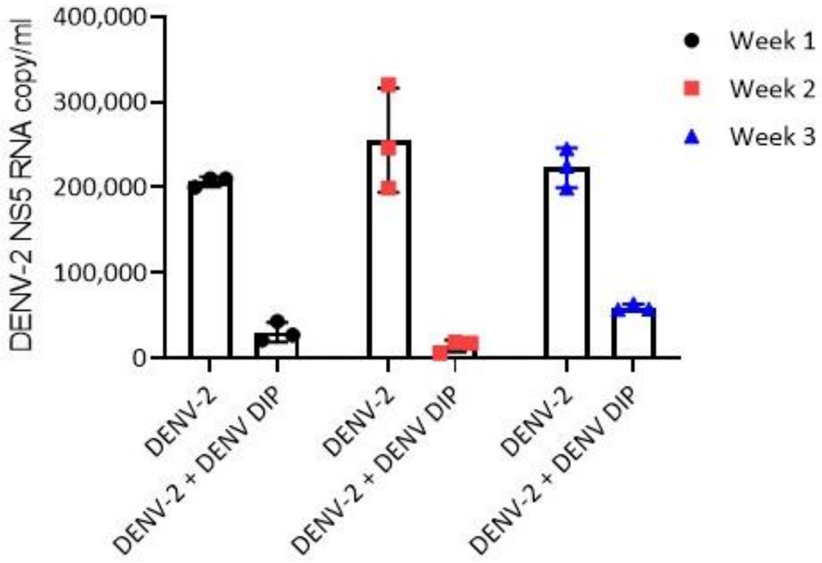

Figure 1S Maintenance of antiviral activity of DENV DIP. The antiviral activity studies of DENV DIPs against DENV-2 replication were conducted in Huh7 cells using the purified DENV DIPs (equivalent to 100 copies of DI-290 RNA/cell) stored at 4 °C for 1, 2 and 3 weeks. Culture supernatants were collected at 72 h post-infection. The levels of viral RNA in culture supernatants were measured by RT-qPCR using primers to the DENV-2 NS5 region. Data are expressed as the mean ± SD from three replicate experiments.

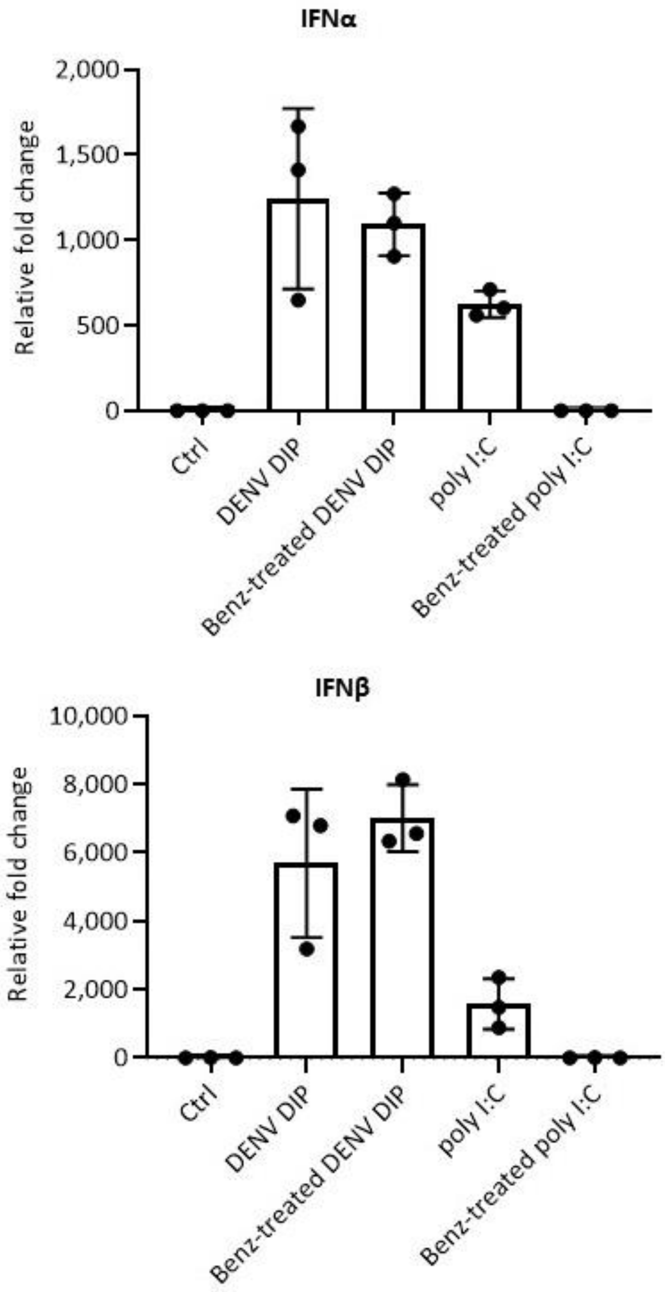

Figure 2S DENV DIP induces IFNα and IFNβ gene expressions. Huh7 cells were treated with DENV DIP (equivalent to 1000 copies of DI-290 RNA/cell), Benzonase (Benz)-treated DENV DIP, poly I:C (5 µg/mL) and Benz-treated poly I:C for 24 h. RNA was extracted and the mRNA levels of IFNα and IFNβ were quantified by RT-qPCR. The data was present as fold change relative to the untreated control cells (Ctrl). Data are expressed as the mean ± SD from three replicate experiments.
